# Experimental Test of Spatial Updating Models for Monkey Eye-Head Gaze Shifts

**DOI:** 10.1371/journal.pone.0047606

**Published:** 2012-10-31

**Authors:** Tom J. Van Grootel, Robert F. Van der Willigen, A. John Van Opstal

**Affiliations:** 1 Radboud University Nijmegen, Donders Institute for Brain, Cognition and Behaviour, Department of Biophysics, Nijmegen, The Netherlands; 2 Max Planck Institute for Biological Cybernetics, Tübingen, Germany; University of Muenster, Germany

## Abstract

How the brain maintains an accurate and stable representation of visual target locations despite the occurrence of saccadic gaze shifts is a classical problem in oculomotor research. Here we test and dissociate the predictions of different conceptual models for head-unrestrained gaze-localization behavior of macaque monkeys. We adopted the double-step paradigm with rapid eye-head gaze shifts to measure localization accuracy in response to flashed visual stimuli in darkness. We presented the second target flash either before (static), or during (dynamic) the first gaze displacement. In the dynamic case the brief visual flash induced a small retinal streak of up to about 20 deg at an unpredictable moment and retinal location during the eye-head gaze shift, which provides serious challenges for the gaze-control system. However, for both stimulus conditions, monkeys localized the flashed targets with accurate gaze shifts, which rules out several models of visuomotor control. First, these findings exclude the possibility that gaze-shift programming relies on retinal inputs only. Instead, they support the notion that accurate eye-head motor feedback updates the gaze-saccade coordinates. Second, in dynamic trials the visuomotor system cannot rely on the coordinates of the planned first eye-head saccade either, which rules out remapping on the basis of a predictive corollary gaze-displacement signal. Finally, because gaze-related head movements were also goal-directed, requiring continuous access to eye-in-head position, we propose that our results best support a dynamic feedback scheme for spatial updating in which visuomotor control incorporates accurate signals about instantaneous eye- and head positions rather than relative eye- and head displacements.

## Introduction

Although saccadic gaze shifts sweep visual images and targets across the retina at high speeds, we perceive the world as stable through a neural process called trans-saccadic integration, or spatial updating. In planning a gaze shift to the next target the gaze-control system compensates for its own behavior to update the visual world [1], but detailed knowledge about the involved signals is still unclear. The mechanisms underlying spatial updating have been studied extensively with the classical open-loop double-step paradigm [2], [3], [4], [5], which requires the programming of two saccades in total darkness in response to brief flashes at different retinal locations. These experiments have shown to invoke adequate spatial updating, as the targeting saccades to the flashed locations are spatially accurate, provided the target flash durations exceed a few ms [6], [7]. For very short flash durations around the first-saccade onset, however, systematic localization errors occur in the direction of the saccade (so-called ‘perisaccadic localization errors’; [6], [7], [8], [9], [10], [11].

**Figure 1:**
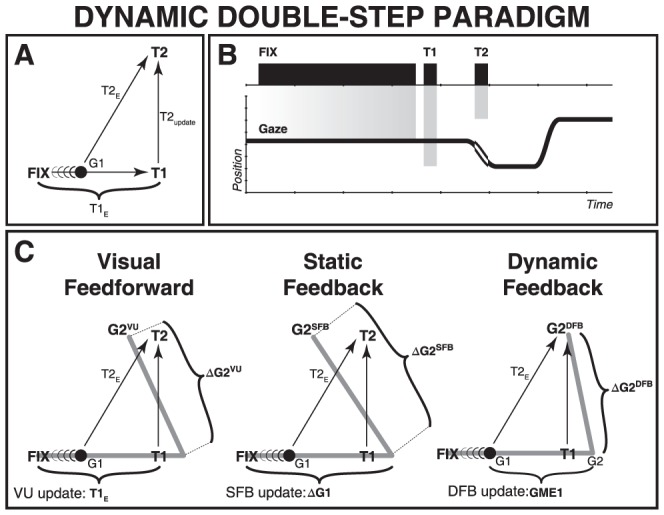
The dynamic double-step paradigm. A) Spatial target configuration. A visual target jumps from the fixation position (FIX) to two successive locations, T1 and T2, where T2 occurs during gaze saccade, ΔG1, towards T1. At T2 onset, the gaze position arrives at G1, and the retinal error is T2_E_. The appropriate motor command for the second saccade, ΔG2, is T2_update_. B) Temporal configuration of the trial. Black bars: target on- and offsets; trace: gaze position (trace highlighted during T2 presentation). Gray bars terminate at target positions. C) Three conceptual schemes to explain target updating after a first gaze shift, ΔG1, that overshoots T1: feedforward visual updating (VU), static feedback (SFB), and dynamic feedback (DFB). The three models make different predictions for the end point of the second gaze shift (G2^VU^, G2^SFB^, and G2^DFB^, respectively). In the dynamic double-step paradigm, only the DFB model predicts accurate behavior, incorporating the first gaze-shift overshoot, and the initial gaze displacement to G1. GME1: motor error for first gaze shift at the time of T2. G2: gaze position at the onset of the second gaze shift.

Various conceptual models, differing mainly in the involved neural transformations, could account for accurate spatial updating. We have recently argued that the dynamic double-step paradigm could in principle dissociate these different models when considering the inherent variability of saccade responses [5] (Fig. 1). In this paradigm the second target flash is presented in midflight of the first gaze shift. As the moment of target presentation is unpredictable, the retinal error of T2 will depend heavily on the current gaze-shift kinematics. Moreover, the eyes move rapidly through space, causing a fast visual streak of target T2 across the retina. Hence, spatial updating under these conditions is a challenging task.

A first possibility is that the gaze-control system only relies on the processing of visual inputs. To plan the second saccade, it could determine the difference, ΔT, between the retinal error vectors (T1_E_ and T2_E_, respectively; feedforward visual updating (VU) model; Fig. 1C, left). The VU model will predict accurate behavior in the static double step, provided the first gaze shift equals the required retinal-error vector of T1, as it does not account for mislocalizations of T1. Yet, behavioral recordings from human subjects have demonstrated full compensation of trial-to-trial variability of responses to the first target, even for very short inter-saccadic intervals [12]. Moreover, neurophysiological experiments have also indicated that visual signals alone cannot account for observed updating behavior. For example, Sparks and colleagues applied microstimulation in monkey superior colliculus (SC) to drive the eyes to a new position within the reaction time of a planned saccade to a visual flash [13]. In this case, accurate spatial updating requires motor feedback without a sensorimotor plan for the elicited, intervening saccade. Indeed, monkeys accurately re-foveated the spatial target location, by compensating for the electrically induced eye displacement. To account for such findings, alternative models propose feedback about the intervening gaze shift, in which case we here distinguish static (Fig. 1C, center) vs. dynamic (Fig. 1C, right) motor-feedback models. In contrast to the VU model, these motor feedback models compensate any mislocalization of the first gaze shift (ΔG1; here illustrated by an overshoot of T1).

In the static motor-feedback (SFB) scheme, the system uses a corollary discharge signal of the entire movement, ΔG1, from FIX toward T1 to prepare the second saccade (Fig. 1C, center). This corollary feedback signal, which reflects the actual movement, and thus any mislocalization of T1, is available to the visuomotor system well before the saccade onset, and has been associated with the phenomenon of ‘predictive remapping’. A seminal series of neurophysiological studies has provided evidence for the presence of corollary discharge signals that represent the upcoming saccade vector (in Frontal Eye Fields (FEF) [14], [15]; in posterior parietal cortex (PPC) [16], [17]; in SC [18]). Cells in these areas show a visual response well before the saccade that brings the stimulus into their receptive field. The visual response appears to predict the visual consequences of the saccade on the retina (hence ‘predictive remapping’), and could underlie trans-saccadic integration and the percept of a stable visual environment. A corollary-discharge pathway has been identified, which arises at the SC, and travels to FEF via the medial-dorsal thalamus [19], [20]. Interestingly, in the dynamic double-step trial of Figure 1 the SFB model predicts systematic localization errors for T2, as it overcompensates the required target update by the movement from FIX to G1 that precedes target onset.

The dynamic motor-feedback (DFB) model employs feedback about the instantaneous gaze shift. In contrast to the SFB and VU models, the saccade goal is a dynamic signal too, and relies on efference copies that correspond to the instantaneous trajectory (in this case, dynamic gaze-motor error, GME1) and movement kinematics (Fig. 1C, right). Eye-position signals have been shown to modulate visual responses of cells in PPC [21], [22] (‘gainfields’). Such signals could be involved in the neural transformation of retinal target coordinates into a craniocentric (or even body-centered) reference frame. It has recently been suggested that such an eye-position feedback signal could potentially arise from a proprioceptive pathway that involves the primary somatosensory cortex [23], [24].

As illustrated in Figure 1C, the three models make different predictions about the final gaze position in the dynamic double-step task: the VU and SFB models predict systematic errors opposing either the direction of the first-saccade error vector, or the saccade gaze shift, respectively. Only the DFB model predicts accurate localization responses.

Most animal studies have measured head-fixed saccades under static visual conditions, in which target flashes were presented with the eyes at rest. In that case the motor-feedback models make identical predictions, and cannot be dissociated. By presenting the second flash *during* the first gaze shift the two motor-feedback models may potentially be dissociated (Fig. 1C). Moreover, by eliciting head-unrestrained gaze shifts the second gaze shift should also incorporate the intricacy of eye-head coordination. This coordination incorporates the contribution of the vestibular-ocular reflex [25]–[29], but also necessitates changes in reference frame for the eye- and head motor systems. For example, head movements to visual targets require the use of eye-position information to update stimuli into a head-centered reference frame [5], [26], [30]. In three dimensions (3D), these kinematic transformations should incorporate the noncommutative and nonlinear properties of fixed-axis rotations (see e.g. the reviews by [31]–[33]).

Human psychophysical experiments have demonstrated that the gaze-control system is equally accurate and precise for static and dynamic double-steps [5], [34]. The present paper extends these results by testing the behavioral responses of head-unrestrained monkeys. Our results support the notion that primate gaze shifts rely on accurate dynamic eye- and head motor feedback signals, as the data can be explained by neither the feedforward VU-model, nor the SFB-model, and are best predicted by the DFB model. Because the results also demonstrate goal-directed head movements, we propose that the primate gaze-control system has continuous access to an accurate eye-in-head *position* signal. Thus, we conjecture that spatial updating involves a world-centered representation of visual targets, rather than a retinal reference frame that is updated by relative gaze displacements.

## Materials and Methods

### Subjects

The experiments were conducted with two rhesus monkeys (*Macaca mulatta*; weight ∼9 kg) that were trained on a visual gaze-shift following and localization task under head-unrestrained conditions [35]. Experiments were conducted in accordance with the European Communities Parliament and Council Directive (September 22, 2010, 2010/63/EU). All experimental protocols were approved by the Ethics Committee on Animal Research of the Radboud University Nijmegen (RU-DEC, ‘Radboud University *Dier Experimenten Commissie*’). Monkeys were pair-housed to promote normal interactive behavior. About 24 hours before the start of an experimental session, water intake was limited to 20 ml/kg. In the experiment, the monkey earned a small water reward of 0.2 ml per successful trial. We ensured that monkeys earned at least the minimum of 20 ml/kg on an experimental day. After an experimental session, water was supplemented to the required minimum amount, if needed, and the animal received additional pieces of fruit. In weekends, the animals' fluid intake was increased to 400 ml daily. To monitor the animal's health status, we kept records of body weight, and water and food intake. Expert veterinarian assistance was available on site. Quarterly testing of hematocrit values ensured that the animal's kidney function remained within the normal physiological range. Our procedures follow the water-restriction protocol of the Animal Use and Care Administrative Advisory Committee of the University of California at Davis (UC Davis, AUCAAC, 2001). Whenever an animal showed signs of discomfort, or illness, experiments were stopped and the animal was treated until the problem was solved.

### Surgical procedures

After the initial gaze-following training was completed, two separate surgeries were performed under full anesthesia and sterile conditions. Anesthesia was maintained by artificial respiration (0.5% isoflurane and N2O), and additional ketamine (IM), pentobarbital (IV), and fentanyl (IV) were administered. In the first surgery, a thin golden eye ring was implanted underneath the conjunctiva to allow for precise eye-movement recordings with the double-magnetic induction (DMI) technique [35]–[38] (see below in section *Head-unrestrained DMI recording of gaze shifts*). In the second surgery, a stainless steel neurophysiological recording chamber (20×12 mm) was placed over the intact skull, centered above the midline, and 2 mm posterior of the interaural line. In addition, one stainless-steel bolt was embedded in dental cement. It allowed firm fixation of the head to the primate chair, needed to prepare the animal for the experiment, and for cleaning purposes. Two additional small bolts embedded in dental cement were used to attach the recording coils needed for the head-unrestrained DMI method, the laser pointer, and the water reward tube (the so-called DMI assembly, described below and [35]).

### Experimental setup

Monkeys were positioned in the center of a completely dark, sound attenuated, anechoic room (2.5 m×2.5 m×2.5 m, all walls lined with 50 mm thick black sound-absorbing foam with 30 mm pyramids, Uxem b.v., Lelystad, AX2250). The monkey was seated in a primate chair that was placed on a platform such that the animal's head was in the center of the room. Body movements were constrained by car seatbelts around the upper arms, and below the chin by a Perspex plate. For liquid reward delivery, a silicon rubber tube was attached to a water-filled receptacle suspended at a height of about 2 m outside the experimental room. The tube terminated on a thin pipe that could be fixed rigidly to the monkey's head (see [35], [36], for details). The light-weight reward system was manufactured such that all fluid was delivered inside the monkey's mouth, regardless head movements, and that the system did not induce any friction or other mechanical obstruction to the head movements.

On the wall in front of the monkey 85 green Light Emitting Diodes (LEDs, λ = 565 nm, viewing angle: Ø 0.2 deg, intensity: 0.5 cd/m^2^ calibrated with a luminance meter, LS100; Konica Minolta, Osaka, Japan) were mounted in a spiderweb-like configuration. The central LED [R,φ] = [0,0] straight in front of the animal could emit a red (λ = 627 nm) or green (λ = 565 nm) fixation spot. Viewing angles of the LED rings were placed at seven eccentricities R ∈ {5, 9, 14, 20, 27, 35, 43} deg. The twelve spokes were placed around the central LED at directions φ ∈ {0, 30, 60, …, 330} deg. We expressed this polar target configuration (R,φ) into a double-pole azimuth (α) and elevation (ε) coordinate system [39] by applying:
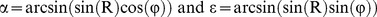
(1)


### Head-unrestrained DMI recording of gaze shifts

Head and eye orientations were measured by magnetic induction techniques, described in detail in our previous work [3]–[6], [35]–[38]. In brief, three orthogonal magnetic fields were each produced by a single-turn pair of coils that were mounted alongside the corners of walls, floor and ceiling, and powered by custom-made audio amplifiers. The magnetic fields alternated sinusoidally at different frequencies (horizontal field: 48 kHz, vertical field: 60 kHz, and frontal field: 80 kHz).

To monitor the head-in-space orientation a small custom-made search coil was mounted on a lightweight assembly that could be rigidly attached with two small bolts to an aluminum holder embedded in a dental cement implant on the monkey's skull [40]. The eye-in-space orientation was measured by our newly developed head-unrestrained double-magnetic induction (DMI) technique (described in detail in [36], and its head-unrestrained extension in [35]–[38]). To that end, a thin golden ring had been implanted underneath the conjunctiva onto the sclera of the right eye. Surgical procedures for implantation of the head holder and eye ring are described in detail in [41]. The oscillating fields produced alternating induction currents in the ring that therefore in turn produced its own secondary magnetic fields, the strengths of which are determined by the orientation of the ring within the primary fields. The small secondary magnetic fields could be measured by a pickup coil that was placed close to and in front of the implanted eye. The signals from the DMI coil assembly, together with the head search-coil, thus provided head- and eye-orientation specific signals.

The signals (head horizontal and vertical, ring horizontal, vertical, and frontal) were fed to five lock-in amplifiers (Princeton Applied Research, PAR, 128A) that decoded the oscillating field signals into DC signals proportional to the measured flux relative to each of the fields (and hence related to eye- or head-in space orientation). Subsequently, the signals were low-pass filtered (150 Hz cut-off, fourth-order Butterworth, custom-built). For offline analysis, these signals were AD-converted at 1017.25 Hz (Tucker Davis Technologies, System 3, TDT 3, RX6, Alachua, Florida, USA), and stored on the computer's hard disk. Before further processing of the data, the signals were digitally low-pass filtered (cut-off 75 Hz, order 50, Hamming-window, linear-phase Finite Impulse Response digital filter). For online monitoring the filtered signals were digitized by a custom-built AD converter (10 bits, 500 Hz, see *Experimental control and timing* section). For further details of the recording technique the reader is referred to [35].

### Calibration

The calibration method was similar to that described in detail in [35]. We here provide a brief outline of the procedure.

### Eye calibration

Monkeys were trained to follow a series of visual target jumps under closed-loop viewing with natural head-unrestrained gaze shifts. The monkey initiated a trial by pressing a handle bar. A randomly selected LED was lit, which extinguished after 600–1100 ms, upon which a different LED was illuminated (for 600 to 1100 ms). This sequence was repeated for a random number of LEDs (between two and six). The last LED in the sequence changed its intensity after a randomly selected duration (600 to 1100 ms). The monkey had to react to this intensity change by releasing the handle bar within 700 ms. The trial was aborted when the monkey released the bar too early (i.e. before the intensity change occurred, or earlier than 100 ms after the intensity change). Monkeys could only detect the small intensity change when foveating the targets. The locations of the dimmed target, together with the raw ring and head signals were used to train two (azimuth and elevation) feed-forward, three-layer neural networks (5 input channels, 5 hidden units, 1 output channel: either response azimuth, or elevation). The networks were trained by back-propagation under a Bayesian regularization algorithm implemented in Matlab's neural network toolbox (Mathworks). The teacher signal was target azimuth, or elevation. The trained networks were subsequently used to calibrate all samples of the raw data signals.

Because the head typically lags the eye when foveating a visual target, many eye-head signal pairs for network training could be extracted from a single localization response. Moreover, since the head contribution to a given gaze shift varies considerably from trial to trial, we could employ this characteristic of the gaze-control system to generalize the DMI signal over a wide range of eye-head gaze positions (90 deg in all directions).

The calibration method relies on a cumulative acquisition of trials recorded over consecutive days. Simulations (described in [35]) and experience with actual data have indicated that about 2000 trials sufficed to provide an adequate calibration with an accuracy within 3% over the entire measurement range.

### Head calibration

In contrast to the eye-in-space DMI signal, the head's search-coil signals have a simple sinusoidal relationship with head orientation, and calibration of the head is therefore relatively straightforward. To that end, the head-fixed laser pointer was aligned with a number of target LEDs by manually directing the monkey's head at the beginning of the recording sessions. Physical constraints impeded the use of the most eccentric LED ring (R = 43 deg), and of the upward (φ = 90 deg) LED at R = 35 deg. For every LED we collected 500 ms of head coil signals, which were subsequently averaged. The monkey was rewarded after each pointing trial. Collection of these head calibration trials took about 3–5 min, and was carried out only once, because of the robustness of the head-coil assembly.

### Saccade detection

Saccades were detected off-line based on velocity and acceleration criteria of the calibrated data. When gaze velocity exceeded 100 deg/s the detection program marked a preliminary saccade onset. When thereafter the gaze velocity dropped below 70 deg/s an offset was marked. On- and offset markings were then fine-tuned towards the nearest drop below zero acceleration. After saccade detection, we examined the main-sequence properties of the gaze shifts (relationship between gaze-shift amplitude vs. duration) and we discarded gaze saccades that fell outside the boundaries determined by twice the standard deviation around the optimal straight-line relationship.

### Experimental control and timing

To ensure millisecond timing precision, the experiment was controlled by a custom-built microcontroller (clocked at 1 kHz). On a trial-by-trial basis, stimulus information was fed to the microcontroller that acted as a stand-alone finite-state machine, which controlled the timing of data acquisition, stimulus selection and presentation sequences, as well as on-line data calibration for window control of the monkey's behavior. An I^2^C (Philips) interface switched the LEDs on and off.

Decision rules for rewarding the monkey were enforced based either on on-line eye or head position signals, on the handle-bar status (up or down), or stimulus timing (on- or offset). Raw analogue eye and head signals were digitized and calibrated online by the trained neural networks (described above) that had been uploaded to the microcontroller.

Experimental properties and conditions were monitored and set by an in-house Matlab (Mathworks, v7.7, Natick, NA, USA) program via a graphical user interface (running on a Dell Precision T3500 PC, Windows XP, Intel, 2.8 GHz) that was connected via a serial port interface (RS232) to the microcontroller. A z-bus interface connected with the TDT system to control data storage on the computer's hard disk.

The monkey's overall behavior was also monitored by an infrared webcam (E-tech, IPCM03), which was connected to a separate PC (Dell Optiplex GX6200, Windows XP, Intel, 2.4 GHz).

A typical recording session lasted about 2.5 hours during which 300–600 correct double-step trials were collected. At the start of a session the head was fixed to allow for fixation of the reward system and eye-head coil assembly. Then, the monkey's head was aligned with the central fixation LED, by using the head-fixed laser pointer (LQB-1–650, World Star Tech, Toronto, Ontario, Canada). Subsequently, the head was released and the monkey performed about 20 trials of central fixation to determine the natural head-offset. The mean offset was subtracted from the laser-guided head calibration [26]. Subsequently, we collected 150 head-unrestrained dimming trials to gather new calibration data (see above). After this initial calibration we started the single-step/double-step experiment.

### Single-step and double-step trials

**Figure 2:**
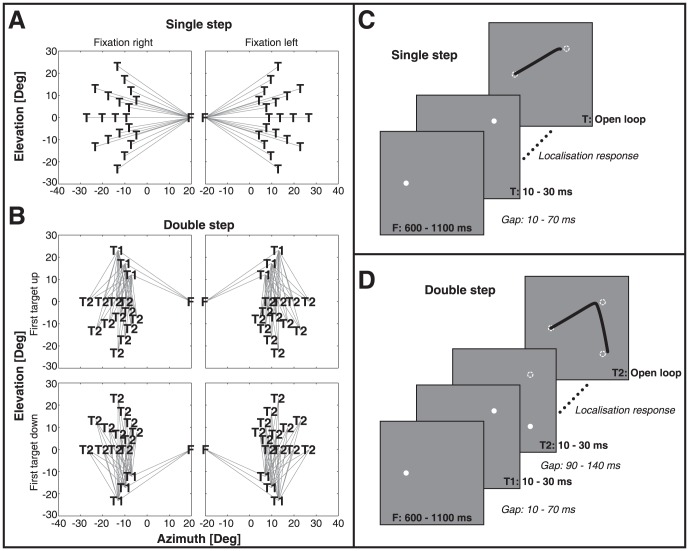
Spatial and temporal layout of targets. A) 40 single-step target locations, F: fixation, T: target. B) Double-step target locations F: fixation, T1: first target, T2: second target. This yields 144 unique double-step configurations. C) Timing of single-step trials. During the gap and the localization period no targets were lit. D) Timing of double-step trials. During both gap periods no targets were lit. The first localization response could have started during T2 presentation. Note that in single-step and double-step trials the last target reappeared after the end of the localization responses (providing visual feedback).

In the experiments, single-step trials and double-step trials were randomly intermingled. Figure 2 shows the spatial (Fig. 2A, 2B) and temporal (Fig. 2C, 2D) events of the single- and double-step trials. To ensure monkeys did not change their strategy, e.g. by waiting for two stimuli to be presented before making a response, the largest proportion of trials were single-step trials (56%). Single-step stimuli (Fig. 2A) contained all target locations used in the double-step trials. The double-step target locations are shown in Figure 2B.

A bright target LED at the initial fixation location announced the start of a trial. Monkeys were required to fixate the LED (8 deg window) and press the handle bar. In single-step trials (Fig. 2C) the dim-lit fixation light (F) was extinguished after a randomly selected period between 600 and 1100 ms. Then, after a randomized gap of 10 to 70 ms, the brief target flash was presented for a duration between 10 and 30 ms in 1 ms steps. In double-step trials (Fig. 2D) the fixation light (F) extinguished after 600 to 1100 ms. After a randomized gap of 10 to 70 ms, the first target was presented at T1 for a duration between 10 and 30 ms. Subsequently, a randomly-selected gap between 90 an 140 ms was followed by a second brief flash at location T2 (target duration in [10, 11, 12, …., 29, 30] ms). Flash durations were set at these brief values, since pilot experiments had indicated that at longer flashes monkeys would often skip the target T1 altogether, and made a saccade directly to T2 (e.g. in our human study, flash durations were 50 ms, see [5]).

Whenever the monkey's gaze shift ended within a 40 deg window around T2, target T2 was presented once more. The target then remained lit until the monkey fixated the final target location (within 2 seconds; window 8 deg). This final closed-loop target was used to ensure that the monkey was able to receive a reward for localizing a visual target, despite a potential mislocalization of the T2 flash in the double-step trial.

### Localization response

The localization response to T2 was identified by the endpoint of the last saccade that had started before the closed-loop T2 target onset. Small correction saccades during the open-loop presentation of T2 were not included in the analysis.

When the monkey made only one saccade in a double step trial, the trial was discarded. In part of these trials, T1 was not fixated at all, and the response was immediately directed towards T2 (15% of double step trials). About the same proportion of trials contained a single curved saccade (16% of double step trials). In these trials saccades were initially directed to T1, but curved towards T2 in midflight. Although these responses were clearly goal directed, it was not straightforward to establish where the response towards T1 stopped, and the T2 directed saccade started. We therefore did not analyze these responses in the present study.

### Static or dynamic double-step trials

When T2 offset was later than the offset of the saccade towards T1, the trial was removed from further analysis, as in these cases the visuomotor system potentially received static visual feedback about the location of T2 before initiating the second saccade. We thus only included trials for which the saccade plan could not be based on direct retinal feedback.

Static or dynamic double-step trials were identified by the amount of movement during T2 stimulation. In static trials this movement did not exceed 0.5 deg (corresponding to foveal fixation), whereas in dynamic trials this midflight movement had to exceed a criterion of 5 deg.

### Data analysis

All off-line data analysis was performed with custom-made Matlab routines.

### Localization error: undershoot-overshoot

To determine localization performance, the signed localization error for each individual trial and response component was determined as:
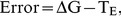
(2)with ΔG the gaze displacement and T_E_ the oculocentric target location (target relative to eye), which equals the gaze-motor error for the saccadic system. Error >0 means the response ended right (horizontal) or down (vertical) from the target. To assess localization errors from an oculomotor viewpoint, they were converted to under- and overshoots. When saccades started right of (horizontal), or down from (vertical) the target, we inverted the sign of the error in Eq. 2. In this way gaze-shift undershoots were always negative, and overshoots positive.

### Linear regression

We quantified localization performance by examining the linear relationship between stimulus location and gaze-response amplitude. The analysis was performed separately for the horizontal (azimuth) and vertical (elevation) gaze-shift components:
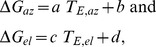
(3)where ΔG is the measured saccadic displacement of the eye in space (for azimuth and elevation components, respectively). T_E_ represents the target location relative to the eye. Parameters *a, b, c* and *d* are regression coefficients, which were found by minimizing the mean-squared error [34]. The dimensionless coefficients *a* and *c* are the response gains, whereas *b* and *d* (in deg) are response biases. Perfect localization corresponds to a gain of 1.0 and a bias of 0 deg. We also determined the coefficient of determination (variance explained by the model) between data and fit (r^2^, with r Pearson's linear correlation coefficient).

### Multiple Linear Regression (MLR)

To quantify the performance of the three different updating models described in the *Introduction* (Fig. 1C), we performed multiple linear regression (MLR) analyses on the second gaze shifts (ΔG2) elicited in the static and dynamic double-step trials. In these analyses, the regression coefficients reveal to what extent the different factors (target locations in initial retinal coordinates: T1_E_ and T2_E_, the full first gaze displacement: ΔG1, or the dynamic gaze-motor error of the first gaze shift: GME1) explained the observed response, ΔG2. As a simple benchmark test against which all updating models could be referred, we also predicted the second gaze shifts in the absence of any updating (no compensation, ΔG2^NC^; target T2 then remains in retinal coordinates). In accordance with the remapping models of Figure 1C, we analyzed the data as follows:
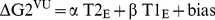
(4a)

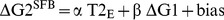
(4b)

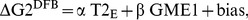
(4c)with α, β and bias the regression coefficients for the respective models. Note that the three regression models had the same number of parameters and degrees of freedom. T2_E_ (T1_E_) is the eye-centered (retinal) representation of T2 (T1) at the time of presentation. For the visual updating model of Eq. 4a, the ideal values are: α = +1, β = −1, and bias = 0 deg (Fig. 1C, left). In case the analysis of Eq. 4a would reveal that α = +1, β = 0, there would be no spatial updating at all (second gaze shift in the direction of the retinal error vector of T2). In Eq. 4b, ΔG1 is the full first gaze displacement vector as used in the static feedback model, for which the ideal values are α = +1 and β = −1 (Fig. 1C, center). In Eq. 4c, GME1 is the dynamic gaze motor error of the dynamic feedback model, and is defined as: GME1 = G2−G1. Here, G1 and G2 are the eye-in-space positions at T2 onset, and the second gaze-shift onset, respectively. Again, the ideal values are α = +1 and β = −1 (Fig. 1C, right).

Note that α = +1, β>−1 in Eqs. 4b–c would correspond to systematic target mislocalizations in the direction of the first gaze shift (e.g., [8], [34]).

To establish whether monkey head movements in the double steps were driven by a gaze-error signal [28], or were instead goal-directed and determined by the appropriate head-motor error signal [26], we analyzed the head-displacement component (ΔH2) of the second gaze shift as function of the gaze-motor error (GME2) and the head-centered motor error (HME2):
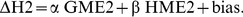
(5)The head-motor error is defined as the location of the second visual target flash with respect to the head at the second head-movement onset. If α≫β head movements are predominantly driven by an oculocentric gaze motor-error signal. Conversely, if β≫α, the head movement would be driven by a craniocentric signal. The latter requires a reference frame transformation of the visual target from oculocentric into head-centered coordinates. Note that ΔH2 was measured until the gaze-shift offset. This head-movement component was typically smaller than the total excursion of the head saccade, as head movements would often continue after gaze had reached the target location, and the vestibular-ocular reflex (VOR) would kick in to drive the eyes back toward the center of the oculomotor range.

### Statistics

We found the optimal regression parameters of Eqs. 3, 4a–c and 5 on the basis of the least-squares error criterion. We then imposed a 2 times SD cutoff criterion after a first regression to remove obvious outliers in the localization responses. In monkey M about 1% of the data points were thus removed. Monkey O was more variable in his behavior, which led to the exclusion of 5–13% of data points for the different stimulus conditions, or response components. This had little effect on the subsequent regression parameters in the analysis, but led to a slight improvement of the overall goodness of fit (r^2^) values.

We then applied the bootstrap method to obtain confidence limits of the fit parameters for the different regression analyses. We created 1000 new data sets by randomly selecting data points from the original data set with replacement. Thus, a given data point could be selected multiple times form the original data set. On each new data set we performed the regression analysis; bootstrapping thus yielded a set of 1000 different fit parameters. The standard deviations in these parameter sets served as an estimate for the confidence levels of the parameters for the original data set [42]. To test whether two fit parameters (for gaze and head-motor error, in Eq. 5, and the parameters for the different models in Eqs. 4a–c) differed significantly (p<0.05), we performed a paired t-test.

To test for a difference between two distributions we applied the parameter free Kolmogorov-Smirnov (KS) statistic on the cumulative distributions.

We compared the coefficients of determination (r^2^) for the different multiple linear regressions Eqs. 4a–c with paired t-tests to decide which of the models explained the data best. The accepted level for significance was p<0.05.

## Results

**Figure 3:**
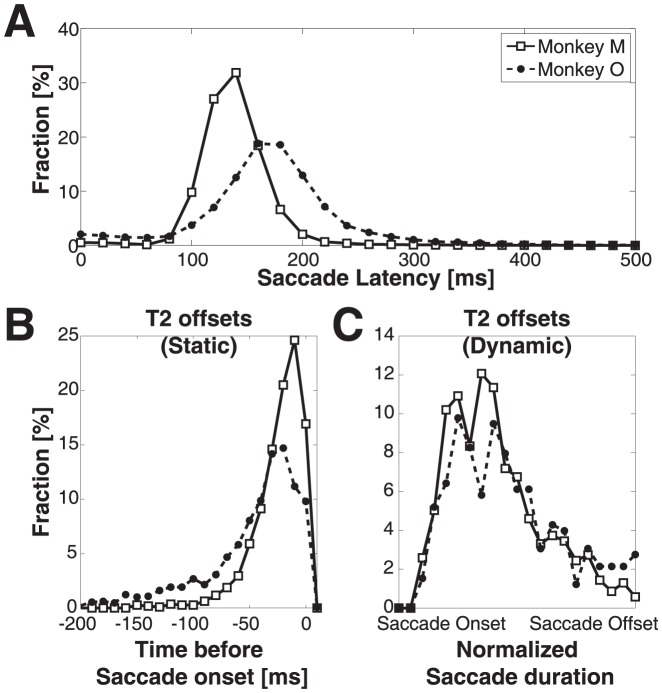
Temporal properties of double-step responses. Result of monkey M is indicated by a solid line with open squares, monkey O by a dashed line with filled circles. A) Latency distributions of first-saccade onsets. Data pooled for all double-step trials. Latency = 0 is T1 onset. B) Distribution of T2 offsets in static trials aligned with first-saccade onset (Time = 0). C) Distribution of T2 offsets in dynamic trials relative to normalized saccade durations. Note similarity of the distributions across monkeys.

By varying the gap between T1 and T2 over a considerable range, we ensured that monkeys would be confronted with static as well as dynamic double-step trials. Figure 3A shows the distributions of gaze-saccade reaction times of monkeys M and O of the first gaze shift towards T1. Although both distributions have similar characteristics (single peaked), monkey O produced somewhat longer reaction times than monkey M (180± s.d. 85 vs. 141± s.d. 30 ms, p≪0.001, two-sampled KS-test). In Fig. 3B we show the distributions of the offsets of the second target flash of static double-step trials for both monkeys. For static trials we required that the second gaze saccade started after the offset of T2. In panel 3C we show the distributions of T2 offset times for the dynamic double-step trials, relative to the normalized first-saccade duration. In these trials the second target was illuminated during the first gaze shift. Note the high similarity of these distributions for the two monkeys.

**Figure 4:**
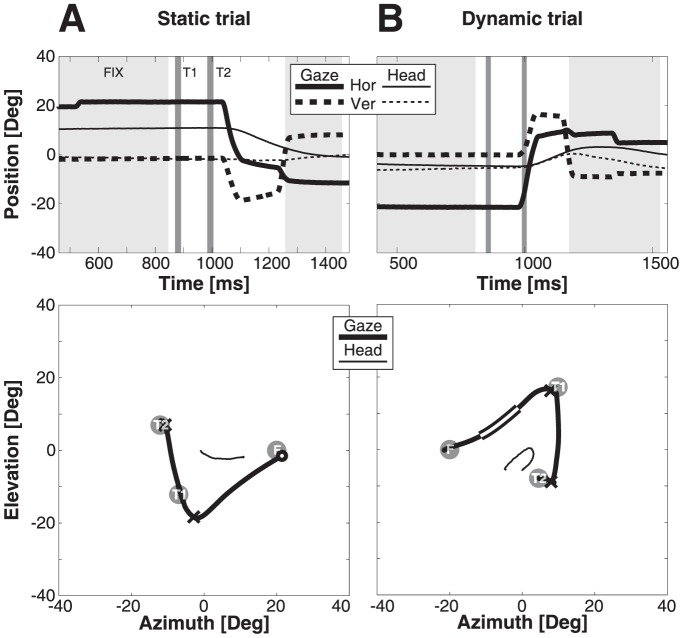
Example responses from monkey M. Gaze (thick lines) and head-position (thin lines) traces for two double-step trials. Top: position as function of time. Solid lines: azimuth; dashed: elevation. Bottom: corresponding two-dimensional trajectories. Target locations are indicated by circles. A) Static trial. Presentation of flashed targets (T1 and T2) are shown in dark gray. Note that T2 is flashed before the saccade. Gaze endpoints of first and second saccade are indicated by ‘X’. Eye position at T2 presentation is indicated by white-filled circle. B) Dynamic trial. T2 is presented during first saccade (the trajectory of gaze displacement during T2 presentation is highlighted by a broader black-white trace in the bottom panel).

Figure 4 provides two representative examples of a static (Fig. 4A) and a dynamic (Fig. 4B) double-step trial of monkey M. The top figures in the panels show the temporal profiles of the horizontal and vertical gaze (bold) and head (thin) movements, whereas the bottom plots give the spatial two-dimensional trajectories. In the temporal traces the timing of the second target (right vertical gray line) can be seen to occur before (static trial) or in midflight (dynamic) of the first gaze shift toward T1. The illumination of T2 during the first gaze shift is also highlighted in the spatial trajectories. In the dynamic trial it can be seen that during the presentation of T2 the eye-in-space makes a considerable movement. Since the target was stationary in space, the gaze shift produced a large visual streak across the right-lower portion of the visual field. In the static trial T2 was presented while the eye-in-space was still fixed. In this trial T2 fell on a visual location about 30 deg left and 10 deg up from the fovea.

**Figure 5:**
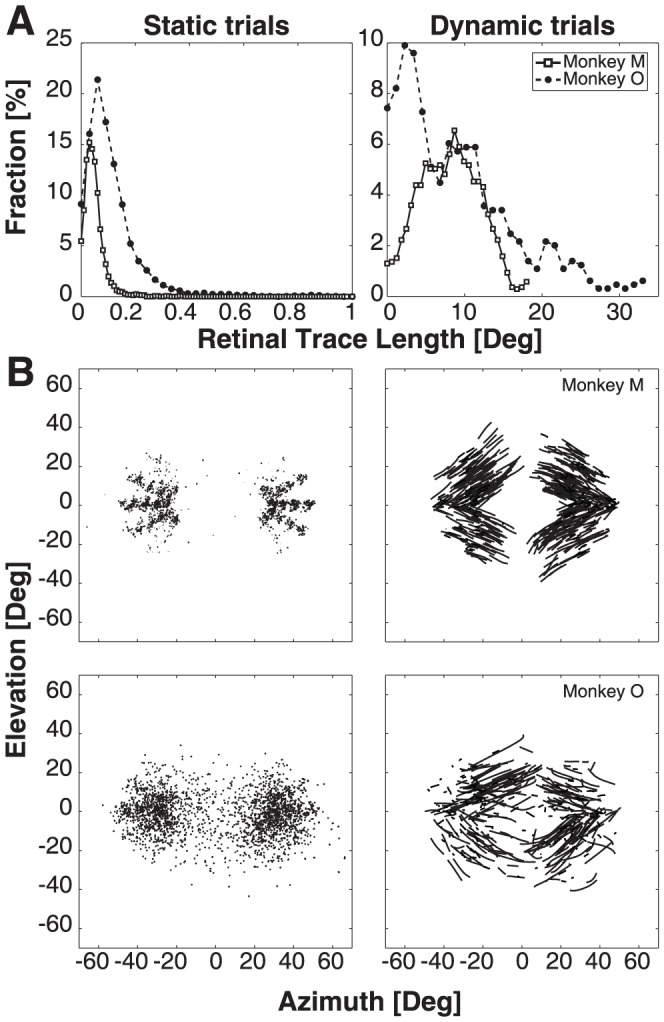
Retinal reconstruction of gaze shifts during T2 presentation. A) Distribution of gaze-movement amplitudes during T2 presentation in static and dynamic trials for both monkeys. Monkey M solid line with open squares, monkey O dashed line with filled circles. Note differences in scales. B) Reconstructed image of T2 locations on the retina. Reconstruction is based on TE = TS – G, with TE the retinal location of the target, TS its spatial location and G the gaze position. The origin of the plot at [0,0] coincides with the fovea. Top row panels: Monkey M, Bottom row panels: Monkey O, Left panels: Static trials, Right panels: Dynamic trials. In dynamic trials the brief stimulus could produce a considerable streak across the retina.

Because the T2 flashes fell on different locations of the retina, had variable durations, and the timing of T2 and gaze-shift kinematics during the flash varied considerably from trial to trial, the visual streak on the retina was unpredictable. To quantify the visual events in the static and dynamic trials, Figure 5A shows the distributions of the retinal streaks during T2 presentation for both monkeys. Note that during static trials the gaze-movement amplitudes remained well below 0.5 deg, so that all potential retinal motion remained within the fovea. During dynamic trials, however, the movement amplitudes were widely distributed, which is due to three factors: variation in T2 flash durations (10–30 ms), to the timing of T2 with respect to the gaze shift, and to the variability in gaze-shift kinematics. In Figure 5B we show the reconstructed amount of visual motion of T2 across the retina during the first gaze shift for static (left) and dynamic (right) trials of both monkeys. During the dynamic trials the visual streak reached values up to, and over, 20 deg, and covered a large part of the retina. Note that the amount of retinal motion for monkey O was slightly larger than for monkey M.

### Localization performance

#### Spatial accuracy and precision

**Figure 6:**
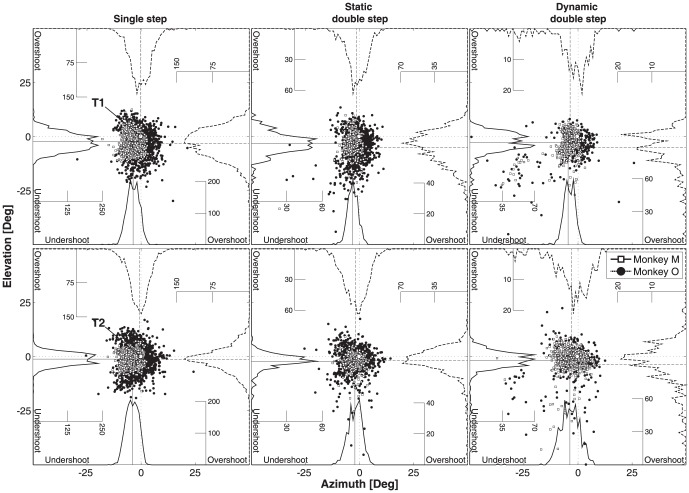
Gaze-localization accuracy and precision in static and dynamic double steps. Data are shown for the three different trial types (single, static, dynamic) for T1 (top row) and T2 (bottom). Localization errors are converted into under- and overshoots with respect to the spatial target location. The center of the panels (x = y = 0, circle and intersection of dotted lines) coincides with the target location. Errors of monkey O: filled dots, monkey M: open squares. Error distributions are presented as histograms (bin size one deg, with frequency axis) at the baseline of each axis. Solid distributions: M. Dashed histograms: monkey O. The solid (M) and dashed (O) lines indicate the mean errors.

Figure 6 shows the response accuracy of both monkeys during the three trial types towards target locations T1 (top row) and T2 (bottom). Note that only T2 is presented dynamically in dynamic double-step trials. In single-step trials T1 and T2 were localized with one saccade, whereas T1 and T2 reflect the same target locations in double-step trials. The accuracy of the gaze responses is expressed as target under- (negative sign) or overshoots (positive; see *Methods*). The mean errors for the horizontal and vertical response components are represented by solid and dashed lines, respectively. It can be seen that responses towards T1 and T2 had, on average, a slight undershoot (between 1.0 to 4.2 deg). Note that the variability of the responses to T1 is higher in the double-step trials than in the single-step trials (see Table 1). This could be related to the fact that in double steps the two motor programs may interfere with each other. For example, averaging could invoke a change in direction and amplitude of the first saccade [4], or the first response could be aborted before its termination. Both effects lead to a larger endpoint scatter. Despite the more variable intervening first saccades, however, monkeys could still localize T2 with reasonable accuracy. The precision (variability) of static and dynamic double step trials showed a slight increase with respect to the single-step responses for the majority of comparisons (p<0.05; KS-test; Table 1). Dynamic double-steps were slightly more variable than static double-steps (p<0.05; KS-test; Table 1). Although the two monkeys differed somewhat in their eye-head coordination behaviors (Figs. 3 and 5), their localization accuracy was comparable, although monkey O was less precise than monkey M (p<0.001; KS-test). Table 1 quantifies the endpoint distributions for the response components, targets and trial types of the two animals.

**Table 1:** Overall localization performance of both monkeys.

T1				T2		
Single step		Static	Dynamic	Single step	Static	Dynamic
				*Mean (±SD)*		
**Monkey M**						
*Azimuth*	−3.80 (±2.82)	−3.20 (±3.03)	−4.45 (±4.45)	−4.21 (±2.86)	−2.11 (±3.16)	−3.62 (±4.67)
*Elevation*	−2.40 (±3.86)	−3.26 (±5.13)	−2.81 (±4.86)	−1.01 (±2.74)	−2.15 (±3.90)	−1.72 (±5.47)
**Monkey O**						
*Azimuth*	−0.37 (±4.62)	−1.22 (±4.83)	−3.56 (±10.54)	−0.98 (±4.55)	−1.83 (±5.19)	−2.79 (±8.80)
*Elevation*	−3.15 (±6.54)	−5.04 (±7.55)	−5.18 (±7.79)	−1.17 (±5.66)	−1.76 (±6.19)	−3.39 (±9.87)

Means and standard deviations (in deg) of gaze-saccade endpoint distritbutions of azimuth (top row) and elevation (bottom row) responses with respect to the target location (origin of Fig. 6) for the first (left) and second (right) target flashes. A positive (negative) mean indicates a target overshoot (undershoot) in that component. Gaze shifts tended to slightly undershoot the target. Comparisons for a statistical difference between distributions were made between the same target components and for the same animal, based on a KS test. Static and dynamic double-steps had significantly more endpoint variability than single-step responses (p<0.05) in the majority of cases. The same holds for dynamic vs. static double steps. Endpoint scatter of monkey O saccades was larger than for monkey M (p<0.001).

#### Linear regression on localization performance

**Figure 7:**
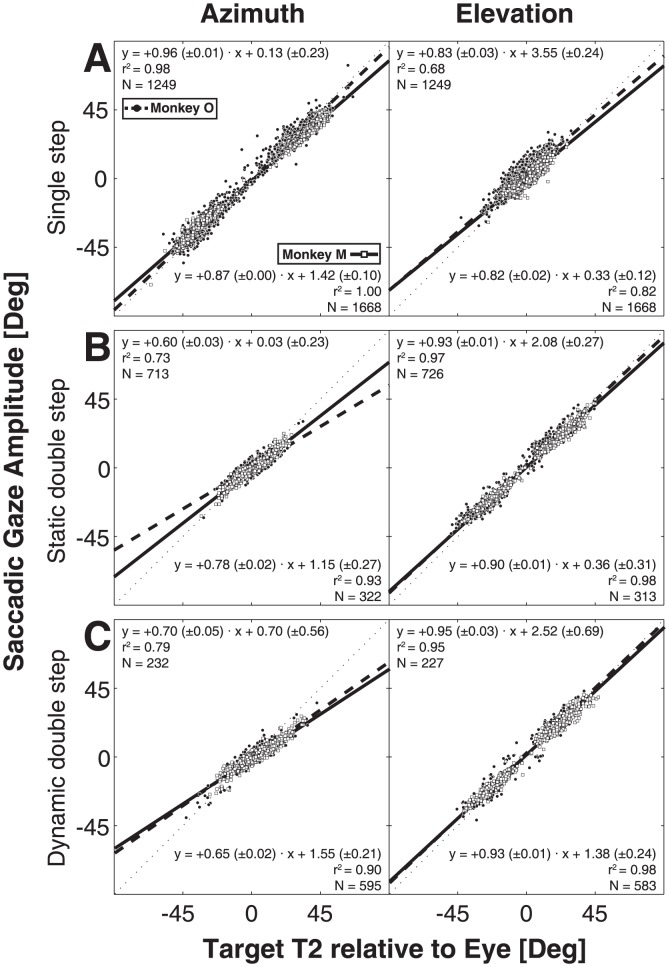
Linear regression of eye-centered T2 location vs. second gaze displacement. Solid line (monkey M) and dashed line (monkey O) are linear fits through the data points (open squares: monkey M, solid circles: monkey O). Thin dotted line represents the unity (x = y) line. Fit values are displayed in the lower-right corner (monkey M) and upper left corner (monkey O) of each panel. A) Regression results for single-step trials. B) static double-step responses. C) dynamic double-steps.

Because the data in Figure 6 suggest that spatial accuracy for the three trial types (single step, and the second gaze shifts in the static and dynamic double steps) was comparable, gaze-shift responses seemed to be goal directed, despite the differences in computational load for the different conditions. To better quantify the monkeys' response performance to the second target flash, we first performed linear regression (Eq. 3) on horizontal and vertical second gaze-shift components of the data for the three trial types. Figure 7 shows the results for both monkeys. The regression lines are shown by a solid (monkey M) and a dashed (monkey O) bold line. The thin dotted diagonal corresponds to perfect localization performance. The analysis indicated that in most cases the slopes of the lines were close to one and the biases near zero deg. This suggests that for all stimulus conditions the gaze-shift responses were driven by the oculocentric target coordinates after the offset of the first gaze shift (indicated by ΔG2^DFB^ in Fig. 1).

### Model predictions

#### Static trials

**Figure 8:**
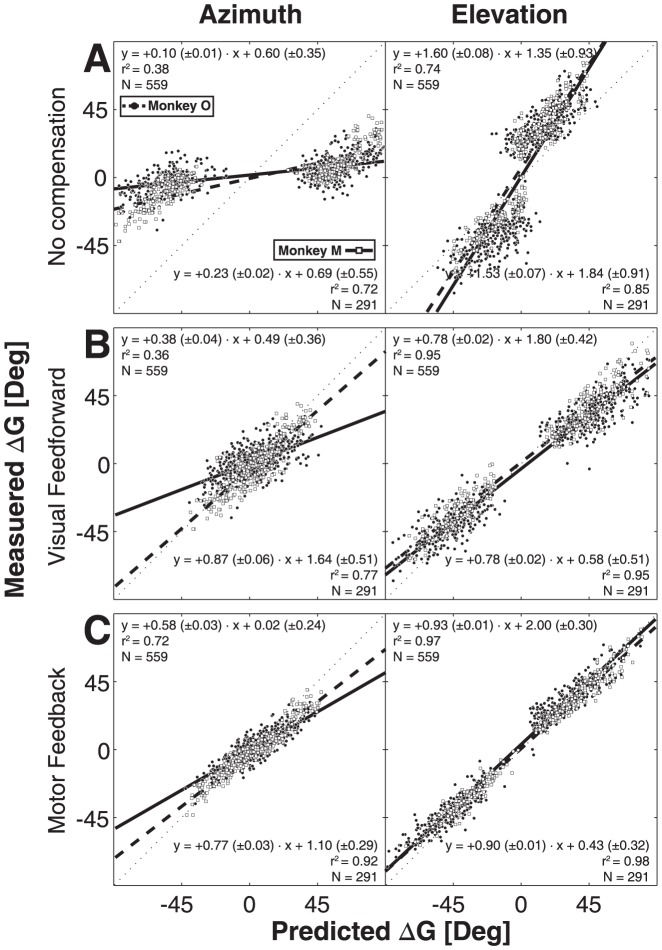
Model comparisons for second saccade vectors in static double-steps. Ideal prediction of a model would align data along the unity line (x = y, thin dotted line). Solid line (monkey M) and dashed line (monkey O) are linear fits through the data points (open squares: monkey M, filled dots: monkey O). Fit parameters are displayed in the lower-right corner (monkey M) and upper-left corner (monkey O) of each panel. A) Predictions of ideal model without spatial updating (Eq. 4a, with α = 1, β = 0). B) Predictions of the feedforward visual updating model (Eq. 4a, with α = 1, β = −1). C) Predictions for motor feedback model that incorporates the first gaze shift (Eq. 4b, with α = 1, β = −1).

As described in the *Introduction*, the static double-step trials yield identical predictions for the two conceptual motor-feedback models (static motor-predictive feedback vs. dynamic motor feedback updating), but the experiment can in principle dissociate the two motor-feedback models from the feedforward visual-remapping scheme and from no updating at all. In Figure 8 we predicted the second gaze-shift responses by applying the ideal regression coefficients of Eqs. 4a–c to the azimuth and elevation response components, and then compared the predicted gaze shifts with the actually measured gaze shifts through linear regression (Eq. 3, with the target coordinates replaced by the predicted gaze-shift coordinates). The data of Figure 8A demonstrate that the scheme without updating (α = +1 and β = 0 in Eq. 4a) is by far the worst model to explain the results. Figure 8B shows the results for the VU model (by applying Eq. 4a to the data with α = +1, β = −1, and bias = 0 deg). Figure 8C shows the result for the motor-feedback models (applying Eq. 4b, with α = +1, β = −1, and bias = 0 deg). From these data we conclude that monkey gaze shifts can be best explained by a model that employs motor feedback, as the pure visual prediction has a far lower coefficient of determination than the motor feedback models (e.g., r^2^ = 0.36 vs. 0.72 for the azimuth components of monkey O). The reason for this difference is that the feedforward VU model (Eq. 4a) does not account for the variable localization errors of the first gaze shifts toward T1 (see e.g. Fig. 6, top-center and right), whereas the actual gaze shifts clearly appear to do so.

#### Dynamic trials

**Figure 9:**
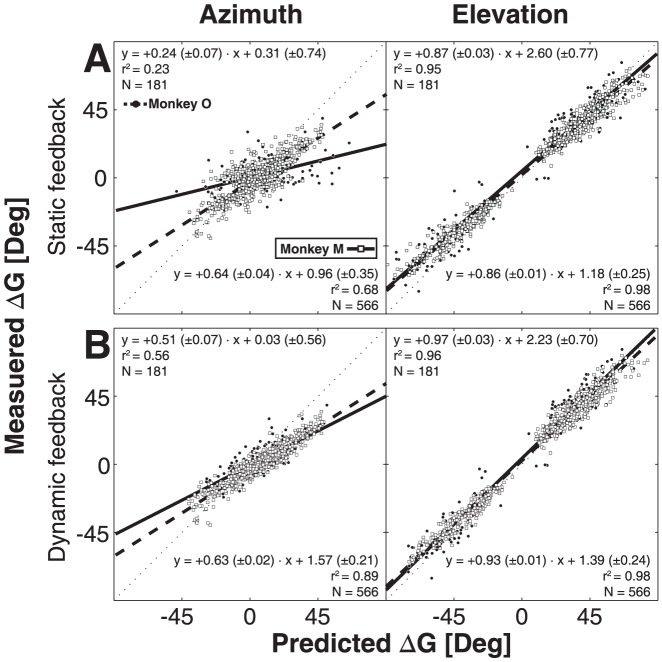
Ideal model predictions for second saccade vectors in dynamic trials. Predictions are made according to static (Eq. 4b, with α = 1, β = −1) (A) and dynamic (Eq. 4c, with α = 1, β = −1) (B) motor-feedback models. Same format as Fig. 8.

To allow for dissociation between the static and dynamic motor-feedback models, the gaze positions at the time of T1 and T2 presentation should differ. We therefore selected trials for which the gaze displacement at T2 onset exceeded five degrees (Fig. 5A, right). In Figure 9 we only show the predictions for the two motor-feedback models (static, predictive feedback (applying Eq. 4b with α = +1, β = −1, and bias = 0 deg), in Fig. 9A; dynamic feedback (applying Eq. 4c, α = +1, β = −1, and bias = 0 deg), in Fig. 9B). As expected from the data in Figure 8, the predictions for the visual models (*no updating,* and *visual feedforward updating,* *Eq. 4a*) were poorer than either motor-feedback model, and are not shown for this analysis. The results show that the dynamic feedback model provides the best prediction of the data for both monkeys. This is especially apparent for the azimuth component (monkey O: r^2^ = 0.23 vs. r^2^ = 0.56), because the major contribution and variability of the first gaze shifts in our experimental design (Fig. 2) was in the horizontal direction.

### Multiple Linear Regression (MLR) analysis

**Figure 10:**
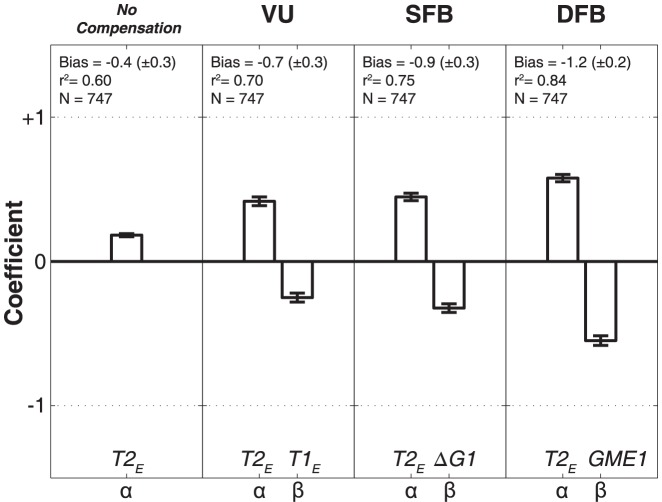
Result of Multiple Linear Regression. The MLR was applied to the updating models of Eq. 4a–c and the no-compensation model. Because first-saccade responses were mainly in the horizontal direction, the elevation components lacked sufficient variation. Accordingly, only responses in azimuth are analyzed. Errorbars indicate 95% confidence intervals. The dynamic feedback model yields coefficients that are closest to the ideal values of with α = 1, and β = −1, respectively. The DFB model also gives the highest coefficient of determination (r^2^), and therefore explains the data best.

In the model predictions of Figures 8 and 9 we described the data with the ideal model parameters, and concluded that the data can be best described by the dynamic feedback model of Eq. 4c, with the strongest discriminative power for the response azimuth components. To quantify to what extent the dynamic change in horizontal gaze position was actually incorporated by the monkey gaze-control system (and thus how far the data departed from ideal), we fitted the azimuth data by applying the MLR analysis of Eqs. 4a–c. The results, pooled for both animals, are shown in Figure 10. Clearly, the high r^2^ value (84% of the variance in the data explained), in combination with the largest β value for the compensatory variable, indicates that the DFB model by far outperformed the other schemes. Nevertheless, the best-fit parameters for the DFB model still deviated significantly from the optimal values of +1 and −1 (see also below, and Discussion).

### MLR on head movements

**Figure 11:**
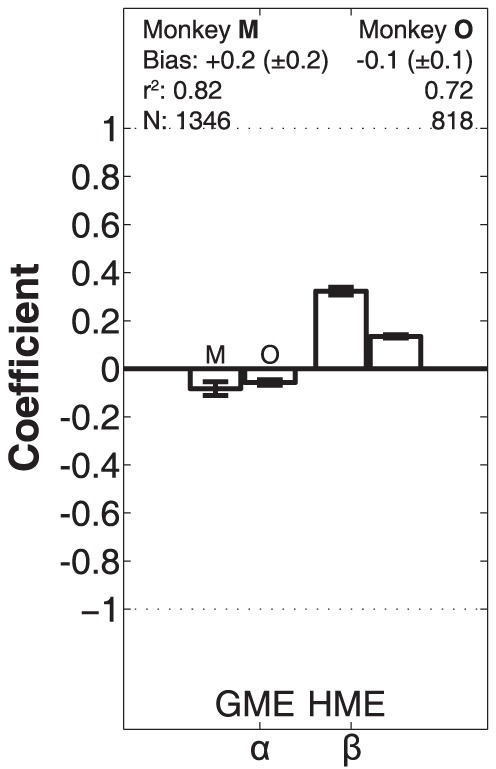
Contribution of gaze- and head-motor error components to the head-movement. The analysis was performed on second gaze shifts in the double steps (measured at between gaze-shift on- and offset), according to Eq. 5 (azimuth components only) for monkey M and monkey O. For both animals, the GME contribution is negligible. Responses are best described by HME, with a gain that differs between the two monkeys, but is significantly different from zero. The coefficient of determination of the regression model is 0.82 for monkey M, and 0.72 for monkey O. Hence, also the head movements toward memorized visual flashes were goal-directed.

Figure 11 shows the results of the head-movement analysis on second gaze shifts for the two monkeys (Eq. 5). Because the head-movements had predominant components in the azimuth direction, we restricted this analysis to the horizontal direction. Although the animals somewhat differed in their gaze-motor strategies, in that the head movements during gaze shifts of monkey O were typically larger than of monkey M (Fig. 5), the head displacements could still be best described by head-motor error for either animal. For both monkeys the head-movement gain at gaze offset was much smaller than one (but differed significantly from zero), indicating that the eyes were eccentric in the orbit at the end of the gaze shift. In contrast, the contribution of gaze-motor error to the head movement was negligible for both animals. In most responses the head movement would continue in the same direction, during which the VOR would drive the eyes back toward the center of the oculomotor range (not shown).

### Effects of T2 timing and flash duration: perisaccadic errors

**Figure 12:**
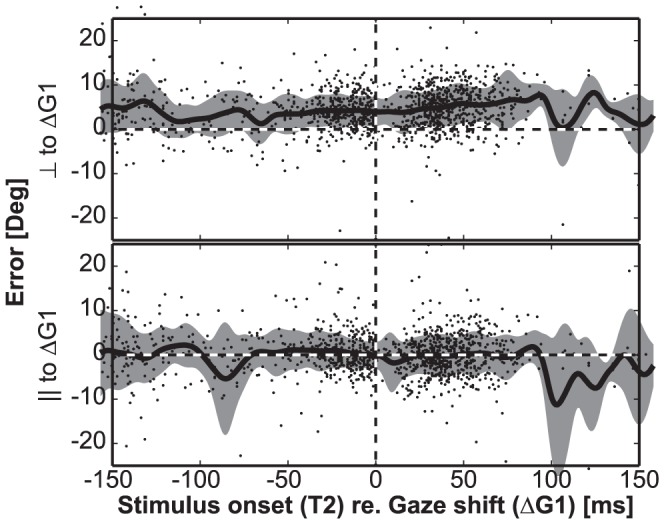
Perisaccadic errors as function of T2 delay relative to first-saccade onset. The perisaccadic localization errors to T2 were computed in the direction perpendicular (top) and parallel to (bottom) the first gaze shift in static and dynamic double-step trials. Data pooled for both monkeys. Solid line: running average; gray shading: standard deviation. In both error components there is no systematic trend as function of the timing of T2 relative to gaze onset.

The open-loop gaze responses in the static and dynamic double steps of the monkeys were endowed with considerable endpoint variability (Fig. 6, Table 1). Moreover, we found that although the DFB model of Eq. 4c could best explain the data (Figs. 8 and 9), the best-fit coefficients of this model deviated significantly from their ideal values (Fig. 10). After applying the model, some of the variability still remained unexplained. We wondered whether the observed endpoint variability would depend on the timing of the second target flash with respect to the first gaze shift. If so, part of the remaining saccade errors might be explained by perisaccadic mislocalization mechanisms, as reported by previous studies [8], [10]. We therefore decomposed the localization error vectors of ΔG2 in a component parallel to the first gaze-shift vector, and in a component perpendicular to the first saccade. According to perisaccadic mislocalization models, only the parallel error component is expected to vary systematically with the perisaccadic flash delay [8]. Figure 12 shows the results for both error components (data pooled for both monkeys). Although the error components scatter over a range of about ±15 deg in both directions, the error patterns did not vary systematically with flash delay for either component.

**Figure 13:**
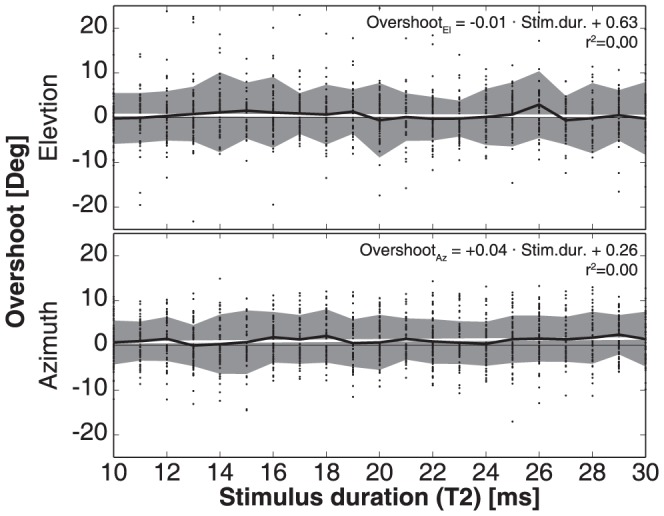
Horizontal and vertical components of the perisaccadic errors as function of T2 duration. Data pooled for both monkeys. There is no trend in the data.

In our experiments we varied T2 flash durations between 10 and 30 ms. We had noted that longer flashes led monkeys to ignore the first target flash altogether, and program a saccade directly to the final target location. These effects were reported also for human double-step behavior, but for longer flash durations [4], [5]. Previous studies indicated that perisaccadic errors depend also on target-flash duration: for longer flash durations peak errors are reduced, for the briefest (<15 ms) target flashes the errors grow [6], [7], [11]. We therefore also verified the potential effect of T2 exposure on the localization errors in our monkey data. In Figure 13 we show horizontal and vertical error distributions ranked for flash duration. If perisaccadic mechanisms would determine the localization errors, the largest errors would occur for the shortest T2 durations. The data show, however, that such an effect did not occur.

## Discussion

The present study is the first to show that monkeys update the spatial location of brief visual targets, flashed in midflight of rapid eye-head gaze shifts in darkness. We analyzed the data within the context of different conceptual models that could account for accurate spatial updating in the classical double-step paradigm: a visual feed forward scheme, relying exclusively on the remapping of retinal stimulus locations (Fig. 1C, left; Eq. 4a), compared to the use of motor-feedback signals (Fig. 1C, center and right). The data show that monkey gaze shifts accounted for potential mislocalizations of the first target in the double step, and thus incorporated the actual motor response (Fig. 8C). This finding excludes updating on the basis of retinal inputs alone. Unlike the classical, static, double-step paradigm, dynamic double-steps also allow for dissociation between predictive motor updating (here termed: static motor-feedback; Fig. 1C, center; Eq. 4b), vs. updating by instantaneous motor performance (dynamic motor-feedback; Fig. 1C, right; Eq. 4c). We conclude that our data favor the latter scheme (Figs. 9B and 10). Finally, our analysis also indicated that the remaining endpoint variability of responses to the second target flash could not be explained by temporal and visual perisaccadic mislocalization mechanisms (Figs. 12 and 13).

### Relation to other studies

Our results show that dynamic head-unrestrained target updating is not exclusive to humans [5], but also occurs in nonhuman primates. Our monkeys were trained on a simple single-target visual-following task, and did not require any specific training for the static or dynamic double-step tasks. This underlines the observation, as reported by [5], that subjects did not realize whether they were in a static, or in a dynamic double-step trial.

#### Absence of perisaccadic mislocalization

Our results (Figs. 6, 12 and 13) indicate that a brief target flash presented immediately prior to, or during a head-unrestrained gaze shift does not induce the systematic mislocalizations that are typically reported in perceptual (head-fixed) visual pointing studies (e.g., [7], [8], [10], [34]). A similar observation was made by [5], [50] for human head-free gaze shifts. These perisaccadic errors, which are in the direction of the first saccade, depend systematically on the timing of the flash with respect to saccade onset (the flash delay), and have been attributed to a sluggish representation of the oculomotor feedback signal (e.g. [8]). We recently suggested that perisaccadic mislocalizations of visual flashes might rather be due to visual factors [7], [11], [34]. First, the size of perisaccadic mislocalizations only weakly relates to the gaze-shift amplitude, where a linear dependence is expected for the filtered motor-feedback hypothesis. Second, perisaccadic errors are virtually absent for auditory-evoked saccades [30], and third, the size of the errors depends on the visual flash duration [7], [46]. Van Wetter and Van Opstal showed that for human subjects flash durations between 5–15 ms induced substantially larger perisaccadic errors than flashes lasting 50 ms [7]. In line with this result, Vliegen, Van Grootel and Van Opstal used 50 ms flashes and showed that indeed the perisaccadic errors around eye-head gaze shifts remained below 5 deg [5].

Hamker and colleagues recently proposed a model to account for the influence of visual, oculomotor, and timing factors on perisaccadic localization errors of head-fixed saccades to brief flashes in darkness [9], [24]. The model assumes that the updating process (hypothesized to take place in the lateral intraparietal cortex) relies on retinal information, on a fast corollary discharge signal of the saccade from the frontal eye fields, as well as on a slower proprioceptive eye-position signal from the somatosensory cortex (see below). The differences in dynamics of these different processes explain the saturation of the influence of first-saccade amplitude. The effect of stimulus duration on the errors follows from the updating decision process. For longer flashes, the decision is made later, and on the basis of more precise information, than for brief flashes.

As shown in Figure 10, the optimal fit parameters for the Dynamic Feedback Model deviated significantly from the ideal values of +1 and −1. A similar result was described for human data [50]. These smaller-than-ideal fit values thus resulted in error patterns that could, however, not be related to perisaccadic mislocalization mechanisms (Figs. 12 and 13). Possibly, most of the remaining variance in the data could be due to random noise in the sensorimotor transformation stages.

Note, however, that even saccades to single visual targets do typically not result in a perfect gain of +1 and a bias of exactly zero deg, as saccades are known to systematically undershoot the target by approximately 10%. Current theories in sensorimotor control assign such behaviors to more advanced sensorimotor strategies that underlie an optimal control principle that minimizes average response errors and response variability, target-acquisition time (e.g. [43]).

It was recently shown that when subjects localize flashes in darkness with eye movements during passive whole-body vestibular stimulation, errors systematically depended on flash duration [6]. For long-duration (100 ms) flashes, saccades were accurate, and fully compensated for the passive-induced head movement. This indicates that the visuomotor system has access to accurate signals about vestibularly induced head rotations (in the absence of neck-muscle proprioception, or a head-motor command), as well as to instantaneous eye position, which changes dynamically during the vestibular nystagmus. Surprisingly, for very brief flashes (0.5 and 4 ms) saccades resulted to be driven exclusively by the flash's *retinal error*, thus ignoring the intervening head and the eye-in-head movement during the saccade reaction time. Because the only difference between long vs. short flashes was the size of retinal motion during the flashes, the authors concluded that visual updating requires firm retinal evidence of self-motion of the eyes through space [6]. They proposed that visuomotor updating involves a (Bayesian) decision process that weighs the evidence and reliability of the relevant cues that relate to stimulus motion and self-motion. The size and direction of the retinal streak, combined with information about eye- and head movements, provides a powerful signal to dissociate stimulus motion from self-motion. However, if the retinal streak is too small (or noisy) to allow for this dissociation, visual updating is canceled, keeping the stimulus in retinocentric coordinates. Note that in this case the localization errors will also be in the direction of the first gaze shift. Interestingly, a similar dependence of stimulus duration was revealed for auditory-evoked saccades during passive vestibular stimulation: longer sounds were adequately localized, whereas very brief (3 ms) sound bursts were kept in their initial head-centered reference frame [44].

In the present experiments, both monkeys employed target updating even for flash durations down to 10 ms (Fig. 13), and irrespective of the flash delay (Fig. 12). Thus, visual motion-detection thresholds in monkeys might be lower than in humans, which could be due to the higher saccade velocities of monkeys (producing larger retinal streaks), or to potentially shorter processing delays in monkeys when compared to humans. Alternatively, the internal programming of actively generated eye-head movements (vs. passive-induced vestibular stimulation) could lower the threshold for visual updating.

It would therefore be interesting to perform static and dynamic eye-head double steps to extremely brief (down to 1 ms, or even less) visual flashes. How, and whether, optimality principles could also account for dynamic double-step behavior has not been studied so far. This topic falls beyond the scope of the present study. Despite its inaccuracies, however, the DFB model was still by far the best spatial updating model to explain the data.

### Neurophysiological implications

#### Visual vs. motor

It may perhaps not come as a surprise that our data discard the feedforward visual updating model (Fig. 8). Earlier neurophysiological experiments had already indicated that spatial updating of a brief target flash also occurs if an intervening saccade is elicited by microstimulation of the midbrain SC [19], [45], [46]. The experiments demonstrated that spatial updating does not require active planning of an eye movement, and seemed to provide strong evidence for the use of motor commands derived at, or downstream from, the stimulated site. Moreover, SC-evoked saccades were even compensated after disrupting eye-muscle proprioception [47].

However, despite those convincing results it cannot be excluded that SC microstimulation may have given rise to a localized visual phosphene that corresponded to the evoked saccade vector, in which case spatial updating of the target flash might still invoke the same feedforward visual updating mechanisms as in a natural visual double step. A problem with microstimulation is that the amount of variability of evoked saccades is typically too small to dissociate the different possibilities [48]. Our behavioral data, however, demonstrate that sufficient variability in first-saccade responses (e.g., Fig. 6) allows for a real dissociation between the measured responses and the visual difference vector (Fig. 8A, 8B).

*Corollary discharge?* Our results point to a spatial updating stage that incorporates instantaneous motor output during gaze shifts to program the next saccade as soon as new visual input becomes available. Neurophysiological recordings in PPC [16], [17], FEF [15], and SC [18] have indicated that the occurrence of a planned saccadic eye movement to a visual target predicts its visual consequences by updating visual neural responses, even before the intervening saccade has started. This *predictive remapping* could potentially underlie the accurate performance of subjects in the double-step paradigm, and the perception of a stable visual world despite rapid saccadic eye movements.

The corollary discharge signal arising from the SC [19], [20] is thought to represent the full desired gaze-displacement vector, encoded as a static, spatially-specific signal in the SC motor map. However, our behavioral data indicate that spatial remapping requires a *dynamic* signal that incorporates only the remaining portion of the gaze shift following target presentation (GME1 in Fig. 1C, right), rather than the full planned saccade vector, ΔG1. As argued by Vliegen et al. [5], [30], systematic localization errors arise when spatial updating uses a corollary signal that represents the full gaze-shift. The center panel of Figure 1C illustrates that the displacement that should be compensated in the dynamic double step (GME1) is overestimated. This overcompensation thus leads to a mislocalization of the target in the opposite direction of the first-gaze displacement. The predicted size of this error is given by the movement from the starting position (FIX) to gaze position G1 at target T2 onset. The data show that such systematic localization errors did not occur, and that the results are best described by a dynamic updating scheme (Eq. 4c).

#### Dynamic updating models

**Figure 14:**
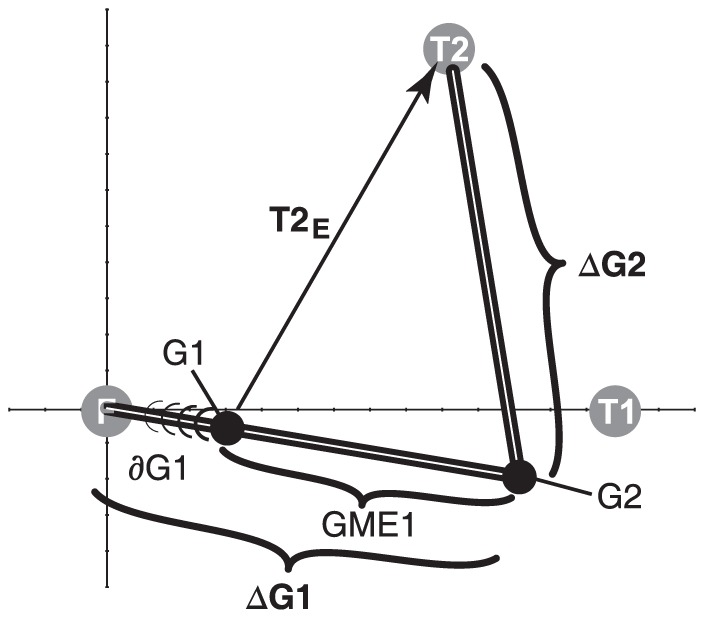
Gaze displacement vs. gaze position feedback. The gaze saccade towards T2 (ΔG2) can either be planned by relying on a dynamic gaze motor-error signal (GME1; Eq. 6b), or by using signals related to current gaze position at stimulus flash onset and second gaze-shift onset, respectively (G1 and G2; Eq. 6a).

Given the millisecond time scale for the underlying neurocomputational processes, as well as the appreciable (visual) delays within the system, accurate spatial updating under dynamic conditions is far from trivial. To successfully perform in the dynamic double-step, the system should have access to (i) the retinal target coordinates of T2 during the gaze shift (T2_E_), and either (ii-a) the instantaneous gaze position (G1), or (ii-b) the instantaneous gaze-motor error during the gaze shift (GME1 = ΔG1 - ∂G1, with ∂G1 the distance of gaze traveled so far, see Fig. 14). From these putative signals, the motor command for the second gaze-shift could in principle be computed in two different ways:
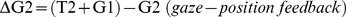
(6a)

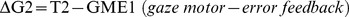
(6b)In case of dynamic gaze-position feedback, the system first transforms the target's retinal coordinates, T2, into a body-centered reference frame by adding gaze position at the time of the flash, G1. At the end of the first gaze shift (with the eye in G2) the second gaze shift is then obtained by subtracting current gaze position, G2, from the stored body-centered target. This model thus assumes continuous availability, and storage, of a gaze position signal during saccades. Such a signal could possibly be constructed from efference copies of the eye-in-head, E_H_, and head-on-neck, H, position signals, as in good approximation: G = E_H_+H.

Alternatively, the updating stage could have continuous access to a dynamic gaze motor-error signal, GME, or rely on gaze-velocity feedback (as suggested in a recent neural network study [49]). Note, however, that GME1 represents an abstract signal that is neither a corollary discharge, nor a proper efference copy signal that could directly drive the eye- and head-motor plants. Figure 14 illustrates the relationships between the different signals in the same spatial reference frame. Note that although the two schemes of Eq. 6a,b are conceptually quite different, they cannot be readily dissociated on the basis of a visual behavioral experiment as in the present study.

#### Gaze-position efference copies?

Which neural stages could carry the signals that implement either of the dynamic transformations of Eq. 6a,b? The dynamic position-feedback scheme utilizes signals directly related to the sensory input (the retinal error, T2), and motor output (the eye-in-head orientation, and head-on-neck orientation). Eye-position signals directly innervate the extraocular muscles, and are found abundantly in brainstem circuitry, e.g. as output of the neural integrator for horizontal, vertical and torsional eye movements [50], [51].

A considerable body of neurophysiological evidence has demonstrated that eye-position signals also modulate the activity of visual receptive fields at various stages within the visuomotor processing chain through so-called multiplicative eye-position gain fields (e.g., in PPC [21]; in midbrain SC [52], and recently also reported for SC activity in head-unrestrained monkey [53]). Such gain modulations could in principle embed, at the neural population level, the neural transformations required for a change in reference frame, such as from eye-centered to head-centered coordinates [22]. Similar modulatory position signals might underlie the control of head-posture. So far, however, the precise role and dynamics of the PPC in these rapid transformations remain elusive. For example, reversible inactivation of the lateral intraparietal area appears to induce relatively minor deficits, such as increased reaction times, rather than the specific mislocalizations that would be expected for a spatial updating deficit [54].

#### Proprioception?

Alternatively, postural signals could be derived from proprioceptive sources, but this seems not a likely explanation. First, although recently a proprioceptive representation of extraocular muscles was found in macaque primary somatosensory cortex, this signal becomes available to the cortex with a considerable delay of several hundreds of ms [23]. As a result, eye-position information reaches the cortex only after the saccade, and is severely low-pass filtered. For that reason, proprioception does not seem to be a good candidate for rapid and accurate spatial updating *during* saccades, when the eyes move with velocities close to one deg/ms. Moreover, SC stimulation-induced saccades were still compensated after cutting the proprioceptive pathway [47]. Finally, intervening microstimulation in the pontine brainstem saccade generator (where burst cells embody the final output of the saccadic system) induced ipsilateral eye-displacements that were *not* compensated by saccades to a flashed target [55]. This latter experiment therefore showed that the oculomotor feedback signals that are used in spatial updating seem to arise *upstream* from the stimulated site, and bypass the proprioceptive pathway.

Taken together, our results support the notion of accurate and fast spatial updating of monkey gaze shifts under challenging visuomotor conditions. We conjecture that the dynamic implementation of spatial updating utilizes accurate signals about eye- and head positions (according to Eq. 6a), rather than an abstract dynamic gaze motor-error signal (Eq. 6b). The required position feedback signals probably arise from high-fidelity efference copies, rather than from proprioceptive sources as found in somatosensory cortex.
